# Adjunctive antidepressive pharmacotherapy in schizophrenia patients

**DOI:** 10.1192/j.eurpsy.2021.110

**Published:** 2021-08-13

**Authors:** M. Zink

**Affiliations:** Department Of Psychiatry, Psychotherapy And Psychosomatics, District Hospital Mittelfranken Ansbach, Ansbach, Germany

**Keywords:** schizophrénia, Depression, antipsychotic, Antidepressant

## Abstract

Depressive symptoms during long-term course of schizophrenia constitute an important and frequent clinical problem. They may occur either as stand-alone major depressive episodes (MDEs) or as part of the schizophrenic negative syndrome. Teatment resistant schizophrenia due to affective deficits results in high subjective burden of disease and a marked subgroup of schizophrenia patients die from suicide. International treatment guidelines strongly suggest offering cognitive behavioural therapy to all patients with schizophrenia. Within pharmacological approaches evidence in favour of second generation antipsychotics exist. The application of mood stabilizers lacks evidence from clinical trials, but is often used in clinical practice. Several antidepressive agents have been administered to depressed patients with schizophrenia and were effective in alleviating both affective and negative symptoms. Treatment outcomes, however, were often limited by side effects and pharmacokinetic interactions, which constitutes the necessity of more easily tolerable pharmacological interventions. Data regarding duloxetine, bupropion, vortioxetine and agomelatine are presented in more detail and discussed within the perspective of multimodal treatment of schizophrenia.

**Disclosure:**

M.Z. received scientific grants from the German Research Foundation and Servier. Speaker and travel grants were provided from Otsuka, Servier, Lundbeck, Roche, Ferrer and Trommsdorff.

